# When is elimination of an infectious disease cost-effective? An analytical framework to guide elimination priorities

**DOI:** 10.1186/s12962-026-00777-2

**Published:** 2026-05-28

**Authors:** Sayara Ahmed, Tara D. Mangal, Timothy B. Hallett, Hugo C. Turner

**Affiliations:** MRC Centre for Global Infectious Disease Analysis, School of Public Health, Imperial College London, London, UK

**Keywords:** Elimination, Economic evaluation, Framework, Value for money

## Abstract

**Background:**

Elimination targets for infectious diseases are increasingly common in global health, yet the economic rationale for pursuing elimination is often assumed rather than rigorously assessed. Existing evaluations frequently emphasise future cost savings or broader economic benefits while overlooking health opportunity costs—the health that could have been gained had resources been allocated elsewhere. This study aimed to develop an analytical framework to investigate when disease elimination generates positive net health benefit (NHB) and to illustrate how key factors interact to shape this assessment.

**Methods:**

We constructed a generalisable analytical framework incorporating ten factors related to intervention and disease costs, intrinsic disease/intervention characteristics, and stakeholder viewpoints and evaluation parameters. The framework was applied to create an exemplar model that showed how these factors jointly influence the NHB of achieving elimination. This was evaluated across wide parameter ranges informed by the literature, using different cost-effectiveness thresholds, discount rates, and time horizons.

**Results:**

The framework revealed distinct regions of parameter space in which elimination yield positive NHB. The cost-effectiveness threshold, discount rates, disease burden, and intervention impact were strong determinants of NHB. In particular, lower thresholds, higher discount rates, and shorter time horizons reduced the likelihood that elimination would generate positive NHB. The framework also showed that elimination may be cost-effective in some settings but not in others, even for the same disease, due to differences in costs, burden, and opportunity costs.

**Conclusions:**

Disease elimination is not always a good investment; its value depends on the interplay between disease characteristics, programme costs, and the health opportunity costs of resource use. The proposed framework provides a transparent, health opportunity cost-based structure for evaluating elimination strategies and supports more robust, context-specific priority-setting on elimination targets in global health.

**Supplementary Information:**

The online version contains supplementary material available at 10.1186/s12962-026-00777-2.

## Background

In the past century, landmark achievements in global health, such as the eradication of smallpox and regional elimination of diseases like malaria, polio, and rabies, have galvanised public health policies and inspired further efforts to target more diseases for elimination. For example, the World Health Organisation’s (WHO) 2021-30 Road Map for Neglected Tropical Diseases (NTDs) set disease-specific criteria and targets for achieving elimination for diseases, including lymphatic filariasis (LF), onchocerciasis, schistosomiasis, and soil-transmitted helminths (STH) [1, 2]. Similarly, following the significant advances in developing treatments like direct-acting antivirals (DAAs), the WHO set global targets to eliminate hepatitis C by 2030 [3].

Two particular types of ‘elimination’ are often discussed in this context: (i) a disease can be eliminated as a ‘public health problem’, which is understood as reducing disease incidence to an ‘acceptable’ level (somewhat above that burden being obviated entirely); and (ii), ‘elimination of transmission’, which refers to the reduction of the incidence of a disease to zero following continuous efforts to prevent reintroduction within a certain geographical region [1, 4]. In either case, “elimination” is generally expected intuitively to be a “good investment” since it terminates a potentially infinite stream of future costs and ill health. However, elimination is an ambitious aim, and the stream of potential benefits may not always justify the cost of it being achieved [5]. Nevertheless, the adoption of such an aim can be driven by political agendas and advocacy, and without an economic evaluation.

The majority of economic analyses in this area have focused on future economic benefits such as reduced costs of illness and intervention costs [5], whilst the health opportunity cost of an intervention (namely the health improvement that could be achieved if the resources required for the intervention were instead used for other healthcare investments) has often been overlooked [5, 6]. Based on these reflections, our study aims to create an framework to understand when intervening to drive prevalence below a certain threshold is potentially “good value”, where that threshold corresponds to the definition of elimination in question.

It should be noted that throughout, we adopt an extra-welfarist approach, consistent with the foundation of cost-utility analysis, where the objective is to maximise contributions to societal health (such as measured in disability‑adjusted life years (DALYs) or quality‑adjusted life years (QALYs)) relative to costs [7]. This contrasts with the welfarist approach (the foundation of traditional cost-benefit analysis), where the efficiency of a health intervention is based solely on the individuals’ perceived value of the welfare that results from it [7]. An implication of this choice is that certain societal benefits that fall outside a health-only perspective may not be captured.

## Methods: an analytical framework

**Fig. 1:**
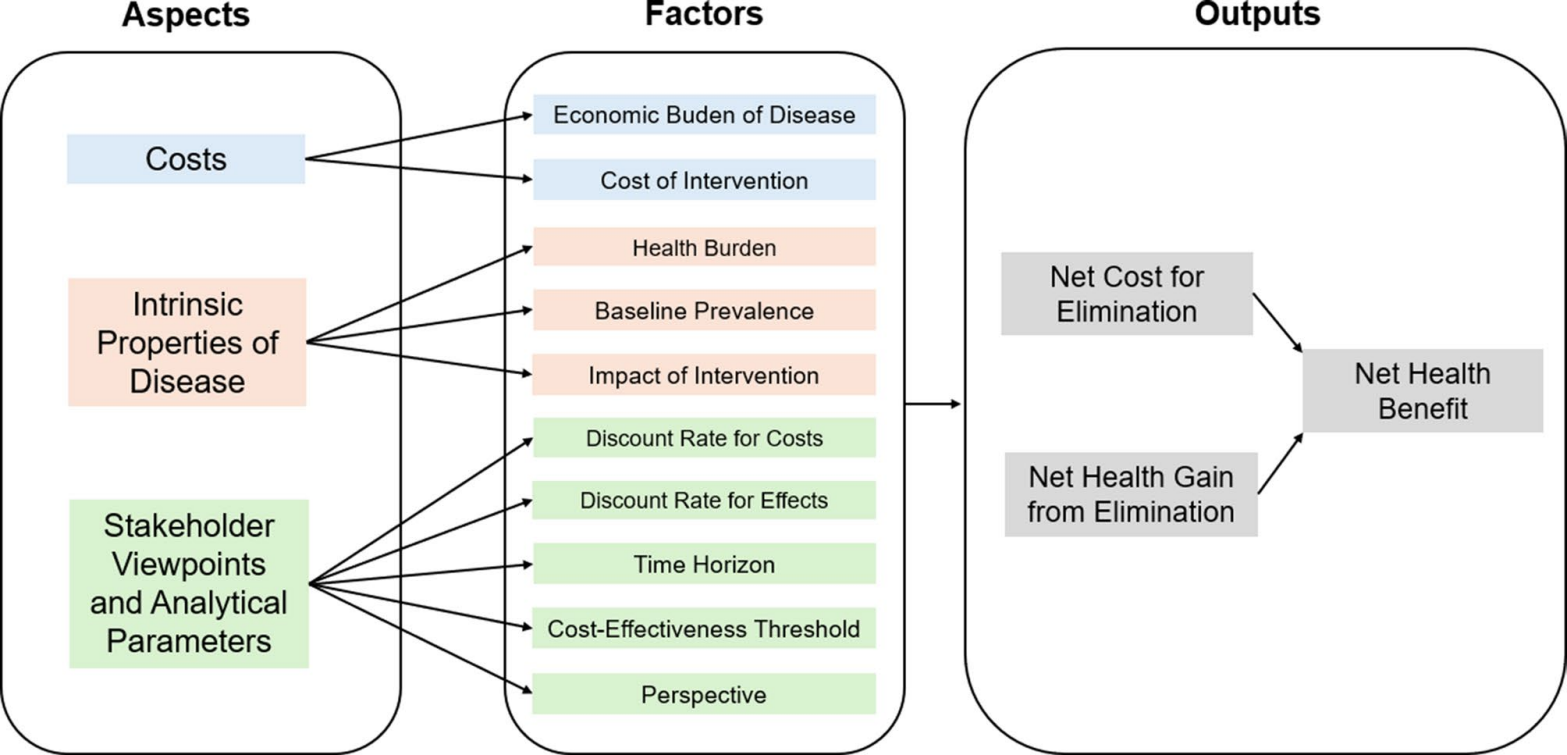
Flow diagram representing the inputs and outputs for the framework

To investigate the conditions under which disease elimination is “good value”, we defined ten factors that characterise the intrinsic costs that result from the disease and the corresponding interventions, the intrinsic properties of the disease, and the stakeholder viewpoints/analytical parameters taken for the analysis (Fig. 1). These were selected to capture the key dimensions that determine the net health benefit - while remaining sufficiently general to apply across diseases and settings. As such, the framework defines the structure of the evaluation, but not the specific parameterisation, which is instead determined in any given application. Critically, although these factors highlighted would typically be considered within standard cost‑utility analyses/are incorporated within established reference cases [8, 9], they are not always made explicit or examined jointly when assessing elimination strategies. 

### Cost factors

The first set of factors in the framework captures the resource implications of elimination strategies and the broader economic burden associated with the disease. Two key components are included (Fig. 1).

The economic burden of disease represents the costs associated with each case in a given year. This may include healthcare expenditures, direct non-medical costs, productivity impacts, and other societal-level consequences, depending on the analytic perspective adopted and data available. As disease prevalence declines over the course of an intervention, this economic burden is expected to fall, generating economic benefits over time. The economic burden of disease may be captured though cost of illness data, but can theoretically also encompass a broader range of economic burden elements beyond traditional cost categories, depending on the analytic perspective.

The cost of intervention reflects the annual resources required to implement the elimination strategy. This may correspond to a single intervention or a package of activities necessary to achieve and sustain elimination.

### Intrinsic properties of the disease and intervention factors

The second group of factors captures characteristics of the disease and the effectiveness of the intervention that jointly determine the scale and timing of health gains.

Within the framework, the health burden represents the total loss of health associated with the disease at the population level. This can be measured using a range of metrics (e.g., DALYs, QALYs). The baseline prevalence of disease captures how widespread the disease is prior to interventions. Together, these parameters determine the total population-level health burden of the disease. They therefore reflect the fundamental health production potential of an elimination program.

The impact of the intervention summarises how rapidly the disease burden is reduced and elimination is achieved under the elimination strategy. High-impact interventions reduce prevalence more quickly, reaching elimination sooner, while low-impact interventions require longer periods of implementation.

### Stakeholder viewpoints and evaluation parameters

The third group of factors captures stakeholder viewpoints and the core evaluative parameters that shape the analysis, rather than intrinsic properties of the disease or intervention.

The discount rates on costs and health effect factors capture time preferences, determining the relative weight placed on near-term versus future costs and benefits. Discounting implies that outcomes occurring further in the future are valued less than those occurring sooner, with the strength of this effect determined by the chosen discount rate.

The time horizon factor determines the period over which costs and outcomes are accumulated.

The cost-effectiveness threshold is a key parameter in economic evaluation, providing the benchmark against which the value for money of an intervention is assessed. Within this framework, it is interpreted as a supply-side threshold [10], representing the health gains that would be displaced elsewhere when resources are committed to an elimination programme. This anchors the evaluation to real resource constraints and reflects the health opportunity cost of funding the elimination program relative to alternative health-generating activities.

Finally, the perspective factor, which is not represented as a numerical parameter but shapes the interpretation of the cost components. A broader perspective, such as a societal perspective, would imply inclusion of additional cost categories—such as productivity losses—within the cost of intervention and cost of illness [11]. This distinction allows the framework to remain flexible while maintaining transparency about which elements of value are included in a given application.

### ‘Determination of ‘Good Value’ and the frameworks output

Within the framework, the determination of whether elimination – for a particular disease, for a particular cost and with a particular viewpoint – is good “value for money” is made by computing the ‘Net Health Benefit’ (NHB) metric [12]. The NHB converts costs into ‘health units’ using the CET (a full derivation of the formula is presented in Supplementary Information):

$$\eqalign{ & {\rm{NHB}}\,{\rm{ = }}\,{\rm{Incremental}}\,{\rm{DALYs}}\,{\rm{averted }} \cr & {\rm{-}}\,\left({\rm{Incremental}}\,{\rm{Costs}\,{\rm{/}}\,{\rm{Cost}}\,{\rm{-}}\,{\rm{effectiveness}}\,{\rm{threshold}}} \right) \cr}$$

In this context, a positive NHB is taken to imply that elimination does represent good value for money, whilst a negative value implies the opposite.

### Exemplar model

To illustrate the use of this framework, an Excel-based exemplar model was developed to evaluate a range of parameter combinations (Table 1 and Supplementary Information). While the framework is intentionally general and not tied to specific metrics or assumptions, the model represents one concrete implementation, requiring the specification of particular functional forms, parameter values, and measurement choices.

The parameter values were informed by reviewing the literature (Table 1). The parameter ranges are not tied to single studies but are intended to span plausible values observed across multiple settings and diseases in the literature. Furthermore, the ranges of values were intentionally broadened to illustrate the two regions of parameter space, and the higher values may not necessarily reflect real-world scenarios.

A no-intervention status quo was used as the comparator of the analysis. In the base case results, a 100-year time horizon is used to ensure that the long-term effects of elimination are fully captured, consistent with methodological guidance recommending time horizons are sufficient to include all relevant costs and benefits [9]. To reflect the interests of decision-makers operating over shorter planning horizons, additional analyses using time horizons of 10, 30, and 50 years are also presented [13].

In the illustrative examples presented, the health burden summarised in terms of DALYs. We assume that the elimination programme achieves a sustained break in transmission once disease prevalence falls below 0.1%. Beyond this point, no further intervention costs or cost of illness are incurred, reflecting cessation of transmission. This assumption is implemented directly in the model structure and is also reflected in the equations presented in the Supplementary Information.

For each combination of costs, the model considered a range of values for the net expected incurred DALYs, disease prevalence, and impact of intervention as the inputs. The entire process was repeated thrice for three different values of the cost-effectiveness threshold (CET), each representing a different setting/income level from Sub-Saharan Africa (SSA).

**Table 1:** Parameter values used in the evaluation of the exemplar model

Parameters	Exemplar Values	Supporting References
Annual economic burden per case of disease i.e. cost of illness (US$)	0, 50, 100, 500, 800	[14–19]
Annual cost of intervention per head of population (US$)	1, 5, 50, 100, 300	[16, 20–22]
Net expected incurred DALYs per case of disease per year	0–1 (0.05 increments)	[23]
Baseline prevalence of disease	2%-10% (2% increments)	[24]
Impact of intervention *	Low, Medium, High	Hypothetical range
Discounting rate for costs **	0%, 3%, 6%	[25, 26]
Discounting rate for effects **	0%, 3%, 6%	[25, 26]
Time horizon	10, 30, 50, 100 years	[13, 25]
Cost-effectiveness threshold (US$) ***	200, 800, 3000	Represents three different settings/income levels from Sub-Saharan Africa***
Perspective	Captured by the cost ranges	[11]

Exemplar values are intended to span the range of all credible values. In the evaluation of the model, a population size of 1 million persons was chosen arbitrarily

*See Additional file 1: Supporting Table S1 for exact numerical values and definitions

**In all the figures presented except Additional file 1: Supporting Figure S2, the discounting rates for both costs and effects were held at 3% [25]. ‘Effects’ refers to the number of DALYs averted

***To capture a range of settings, the values for CET were calculated as approximations of half the 2024 GDP per capita figures for Burundi (the lowest in SSA), the entire SSA, and South Africa (the highest in SSA) [10, 27]

## Results: observations from the multidimensional framework

**Fig. 2:**
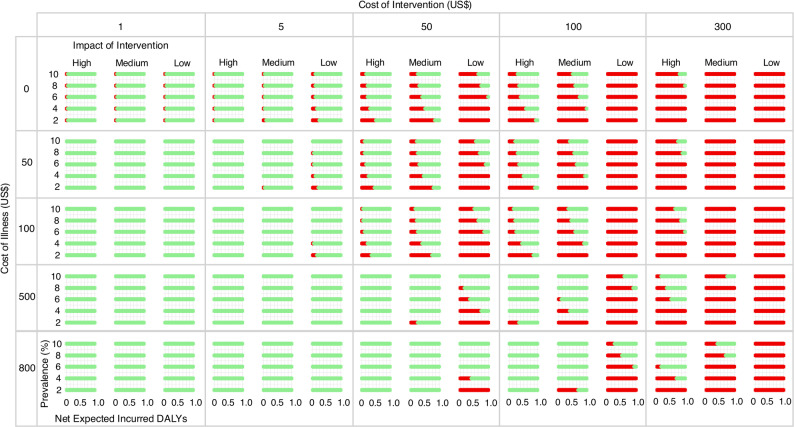
A 5-dimensional graph demonstrating the effect of varying the parameters within the framework: results based on a CET of US$ 800 and a time horizon of 100 years. Green represents a positive NHB (i.e., ‘good value’) and red corresponds to a negative NHB (i.e., ‘not good value’)

The output of the framework are illustrated in Fig. 2, which shows the regions of positive (green) and negative (red) NHB values for each set of parameter values. Each panel represents a distinct combination of disease characteristics, intervention impact, and evaluative assumptions, illustrating how these elements interact to shape conclusions about value for money. The regions of ‘parameter space’ where elimination is good value can thus be visualised.

In general, with higher costs, lower health impact, and lower CET, elimination often does not represent good value for money. When one or more of these conditions applies, a higher prevalence of disease and impact of intervention can shift elimination towards good value.

This figure uses a CET of US$ 800 but the corresponding figures for other CET values are given in Additional file 1: Supporting Figure S1. The comparison between these figures shows that CET is a very strong determinant of the size of the region of parameter space where elimination is good value. With lower CET values, in order for elimination to be cost-effective, greater health benefits must be produced at lower costs. This is pertinent as, despite the widespread concerns/criticism surrounding the use of 1 to 3× GDP per capita cost-effectiveness thresholds, they are still used in the literature [28]. Such cost-effectiveness thresholds run the risk of overestimating the NHB and promoting interventions that may in reality, be bad value when calculated based on a threshold reflecting the health opportunity cost.

The impact of different combinations of discount rates for costs and effects is illustrated in Additional file 1: Supporting Figure S2. It was observed that increasing the discounting rate for costs makes elimination more likely to be bad value. This is likely because the future projected cost savings are weighed less with increasing discounting rates. Similarly, increasing the discounting rate for effects makes elimination less likely to be good value.

The impact of the assumed time horizon on the framework output is highlighted in Additional file 1: Supporting Figure S3. This highlights that elimination is less likely to be found to be good value when stakeholders are interested in a shorter time horizon. This demonstrates that stakeholders will often need to consider long-term horizons in order for elimination to be good value, as it takes time for the long-term benefits to outweigh the investment required. The costs of illness and intervention costs depend significantly on the stakeholder’s perspective. The cost of intervention can be higher if the perspective adopted is broader [11]. For instance, the costs associated with treatment at an outpatient clinic from the healthcare provider’s perspective would include medical costs paid by the healthcare provider (such as for drugs and consumables). Whereas a societal perspective would also capture the cost incurred by the patients to travel to the clinic, their out-of-pocket costs and the value of their lost productivity, increasing the cost associated with the treatment visit. The same principle would apply to what costs are included within the cost of illness parameter [11]. The impact of adopting a broader perspective on the net health benefit (NHB) can therefore vary. On one hand, a broader perspective could increase the averted cost of illness, generating greater cost savings associated with the intervention, thereby increasing the NHB. Conversely, the NHB would decrease if the increase in the cost of intervention outweighs that of the cost of illness when using a broader perspective. This relative impact and direction of the change on the outcome will therefore depend on the context of the study and the intervention being investigated [11].

While several patterns are unsurprising when considered in isolation, the value of the framework lies in demonstrating how strongly their influence depends on the broader configuration of parameters. Taken together, the results highlight how the framework can be used to explore the boundaries between scenarios where elimination is likely to be cost-effective and those where it is unlikely to be justified, rather than to generate a single universal recommendation. Importantly, several factors that are individually unfavourable to elimination can jointly be offset by others, highlighting the risk of drawing conclusions from single parameter comparisons in isolation. These observations are particularly relevant for decision‑makers considering elimination in heterogeneous epidemiological and financing contexts.

## Discussion

Our framework illustrates how different economic considerations shape the relative value of elimination under alternative assumptions. The results demonstrate that disease elimination cannot always be assumed to represent good value for money but instead depends on how a number of factors interact to determine its economic favourability. Crucially, the framework emphasises the need to account for health opportunity costs when estimating the projected value of elimination. This is relevant because, although several studies have presented economic evaluations of elimination strategies for specific pathogens, the net health benefit framework has not been applied in this area. Instead, many analyses rely on incremental cost‑effectiveness ratios (ICERs), cost–benefit ratios/return on investment, or estimates of broader economic benefits or averted health‑system costs [29–31]. Furthermore, elimination is often simply assumed to be an appropriate goal without any economic evaluation. To strengthen the analytical foundations of these important decisions, we provide a generalisable framework onto which all evaluations of elimination could attach, and which makes the health opportunity costs the central benchmark.

The total health opportunity costs of the programme will be influenced by the chosen CET as well as the discount rate for the effects. These are often informed by international/national guidelines and assumed to be constant when projecting into the future (such as a 3% discount rate being commonly used within global health [26], or the CET being based on 0.5 times the local GDP per capita [28]). However, in reality, they will vary across different settings and over time and ideally should be based on country-specific studies/data. This is an important area for future research to refine these values. The source of financing can be an important consideration when interpreting cost‑effectiveness results. Donor funding can ease domestic budget constraints, which may lead to the perception that domestic cost‑effectiveness thresholds are less relevant for donor‑financed interventions. However, donor funding does not remove opportunity costs at the global level—particularly in a context of constrained and declining development assistance. Therefore, considerations of health opportunity costs in this context remain relevant even for interventions financed by donors.

The results of the framework also highlight the critical importance of context-specific decision-making. The same elimination strategy may represent good value in one setting but not in another due to differences in disease burden, intervention costs, epidemiological conditions, and available resources. The framework also underscores the importance of time horizons and discounting assumptions. Elimination strategies often involve substantial upfront investment with benefits accruing over long periods. Short planning horizons or high discount rates can undervalue these long-term gains, potentially biasing decisions against elimination.

It is important to note that the particular Excel exemplar model we present in this paper is only one possible implementation of the underlying analytical framework we have proposed. Whilst this implementation may not be suitable for all possible pathogens and proposed interventions, the underlying framework it represents does aim to be generalisable to all elimination programs. To our knowledge, this is the first such framework to be presented.

### Mapping diseases to the framework

It is possible to map the approximate locations of certain diseases within this framework to assess the case that elimination would be good value for money. We provide select examples below:

In the case of malaria elimination in the Asia-Pacific region, the prevalence was estimated to be ~ 2% in 2021 [30]. The cost of mass drug administration (MDA) was estimated to be US$ 13 per person [30] and the CET of the region is approximately US$ 4,600 [27]. According to the framework for a high CET (see Additional file 1: Supporting Figure S1b), and a cost of intervention of less than US$ 50 per capita per year, it would be potentially good value to eliminate malaria in the Asia-Pacific. Again, this conclusion is supported by a 2020 investment case promoting the prioritisation of continuing malaria elimination efforts [30].

LF is one of the NTDs targeted for elimination through the Global Programme to Eliminate Lymphatic Filariasis (GPELF). The average cost of illness for LF from the healthcare provider’s perspective is typically low (< US$ 5 per year) [32, 33]. Our framework shows that for a disease with a relatively low DALY burden per case, such as LF (with a disability weight of approximately 0.11 [23]) and a low cost of illness, a low intervention cost would be necessary for elimination to be good value. It should be noted that the estimated cost of illness for LF from a societal perspective was much higher [15], highlighting the potential for larger economic benefits when using a broader perspective.

### Limitations

The goal of this framework was to highlight key features that need to be considered when evaluating elimination programmes and the corresponding multidimensional parameter space. To reemphasise, our exemplar model was used as a case study to illustrate the framework. Hence, the analytical framework itself could be used with more advanced disease-specific models capturing transmission dynamics and population heterogeneity, thereby producing non-uniform rates of prevalence reduction over time as well as accounting for stochasticity.

In addition, the model we used to illustrate the analytical framework did not reflect the non-linearity in costs that would be expected from escalating costs when nearing elimination [34]. The impact of the interventions can also change over time to become less impactful if the interventions do not become more targeted towards high-risk populations when nearing a low prevalence of disease. A further limitation of the model is its ambiguity regarding morbidity and mortality with the DALY calculations and the cost of illness. For simplicity, the model considered average values per case and did not distinguish between morbidity and mortality. We attempted to capture both scenarios by using a wide parameter range.

It is important to note that the exemplar model also does not formally capture the difference between elimination as a public health problem and interruption of transmission. It should be noted that the model assumed that the intervention costs stopped once the elimination threshold had been achieved and did not account for the continuing cost of suppression activities, ongoing surveillance and/or the continuing cost of activities to prevent reintroduction. Further investigation into the relative benefits and trade-offs associated with sustaining control measures to maintain a low disease incidence (consistent with elimination as a public health problem) versus the increased cost and subsequent effects of breaking transmission is required.

Additionally, the cost of intervention is related to the population, reflecting preventative programmes. In contrast, for therapeutic interventions, the cost would relate to the number of cases, with the cost reducing in line with the decrease in prevalence over time. That said, the overarching principles illustrated by the framework would apply to both scenarios.

Finally, it is important to note that several potentially important sources of value associated with disease elimination are not modelled explicitly in the illustrative analysis. Such effects might, in other applications, include impacts on antimicrobial resistance, spillover benefits to caregivers and households, equity considerations, and broader societal effects (such as averting diseases disproportionately affecting children). Without including these effects, estimates may understate the total societal value of elimination in settings where such benefits are substantial. The framework itself, however, does not exclude these additional impacts from being accounted among the identified factors when data allows – they would simply add to the assumed ‘health gains’ or ‘economic burden’. Similarly, the objective of good value can also be reshaped to fit the context: for instance, approaches such as distributional cost-effectiveness analysis [35] could be used to incorporate the appropriate consideration of health equity. In addition, when conducting an evaluation of elimination strategies, any relevant sources of value not explicitly quantified should always be clearly identified and discussed alongside the quantitative results, supporting a more comprehensive and nuanced interpretation by decision-makers.

## Conclusions

We have proposed an intuitive framework that identifies the key features that need to be considered regarding the value of elimination and highlights the interlocking sets of factors that determine whether disease elimination can be considered good value for money. In many cases it can be, but, equally, there are many circumstances where elimination would not be, such as an expensive intervention for a disease with a low health burden. As these factors include intrinsic properties of the disease, cost of intervention and disease, and stakeholder/analytical parameters for the evaluation, the apparent ‘value for money’ will depend on the local and prevailing circumstances: elimination can be a good value investment in one location or at one time, but not in others, despite the pathogen and the efficacy of the interventions available being identical. The framework also highlights the need to consider health opportunity costs in the context of the evaluation of elimination programmes and that not doing so may overstate their economic value. Overall, this work highlights that the economic value of disease elimination is a complex and multidimensional problem.

## Supplementary Information

Below is the link to the electronic supplementary material.

Supplementary Material 1

## Data Availability

All data relevant to the study are included in the article or uploaded as supplementary information.
