# Supplementary material for: When is elimination of an infectious disease cost-effective? An analytical framework to guide elimination priorities
Source: Cost Eff Resour Alloc. 2026 May 28;24:98. doi: 10.1186/s12962-026-00777-2 (PMC13411859; doi:10.1186/s12962-026-00777-2)
Supplement: Supplementary file 1 — Supplementary Material 1 [file 12962_2026_777_MOESM1_ESM.docx]

**ADDITIONAL FILE: SUPPLEMENTAL INFORMATION**

**Model equations**

For the ‘Intervention Arm’:

$$Total Cost (A_{1})=\sum_{t=0}^{T} [C\times N\times p\times\left( 1-i \right)^{t}\times\left( 1-d_{c} \right)^{t}] + \sum_{t=0}^{Y} [c\times N\times\left( 1-d_{c} \right)^{t}]$$

$$Total DALYs Averted (B_{1})=\sum_{t=0}^{T} D\times N\times p\times{(1-i)}^{t}\times{(1-d_{e})}^{t}$$

For the ‘No Intervention Arm’:

$$Total Cost (A_{2})=\sum_{t=0}^{T} C\times N\times p\times{(1-d_{c})}^{t}$$

$$Total DALYs Averted \left( B_{2} \right)=\sum_{t=0}^{T} D\times N\times p\times{(1-d_{e})}^{t}$$

$$Net Health Benefit=\frac{[\left( B_{2}-B_{1} \right)-\left( \frac{A_{1}-A_{2}}{U} \right)]}{N}$$

*where, C = Annual cost of illness per case of disease,*

*c = Annual cost of intervention per head of population,*

*D = Net expected incurred DALYs per case of disease per year,*

*d_c_ = Discounting rate for costs,*

*d_e_ = Discounting rate for effects,*

*i = Impact of intervention,*

*N = Population size (at time=0),*

*p = Baseline prevalence of disease (at time=0),*

*T = Time horizon (years over which the calculation is performed),*

*t = Time (years)*

*U = Cost-effectiveness threshold,*

*Y = Duration of intervention (i.e., total number of years in which the intervention cost is applied - see Supporting Table 1 for further clarification).*

**Supporting Table 1. Duration of intervention.** *The year until which the intervention cost is applied (the duration of the intervention) is calculated using different values of baseline disease prevalence and the impact of intervention, keeping all other parameters constant. The ‘impact of intervention’ parameter determines the magnitude at which the base case prevalence will decay over the years. Three values for the impact of intervention, 0.1, 0.3, and 0.5, are used since they correspond to a plausible range of intervention years used in real-life elimination strategies, i.e., 5-40+ years. ‘Low’ impact of interventions would take longer to reduce the prevalence of disease, requiring more years of implementing an intervention, and vice versa.*

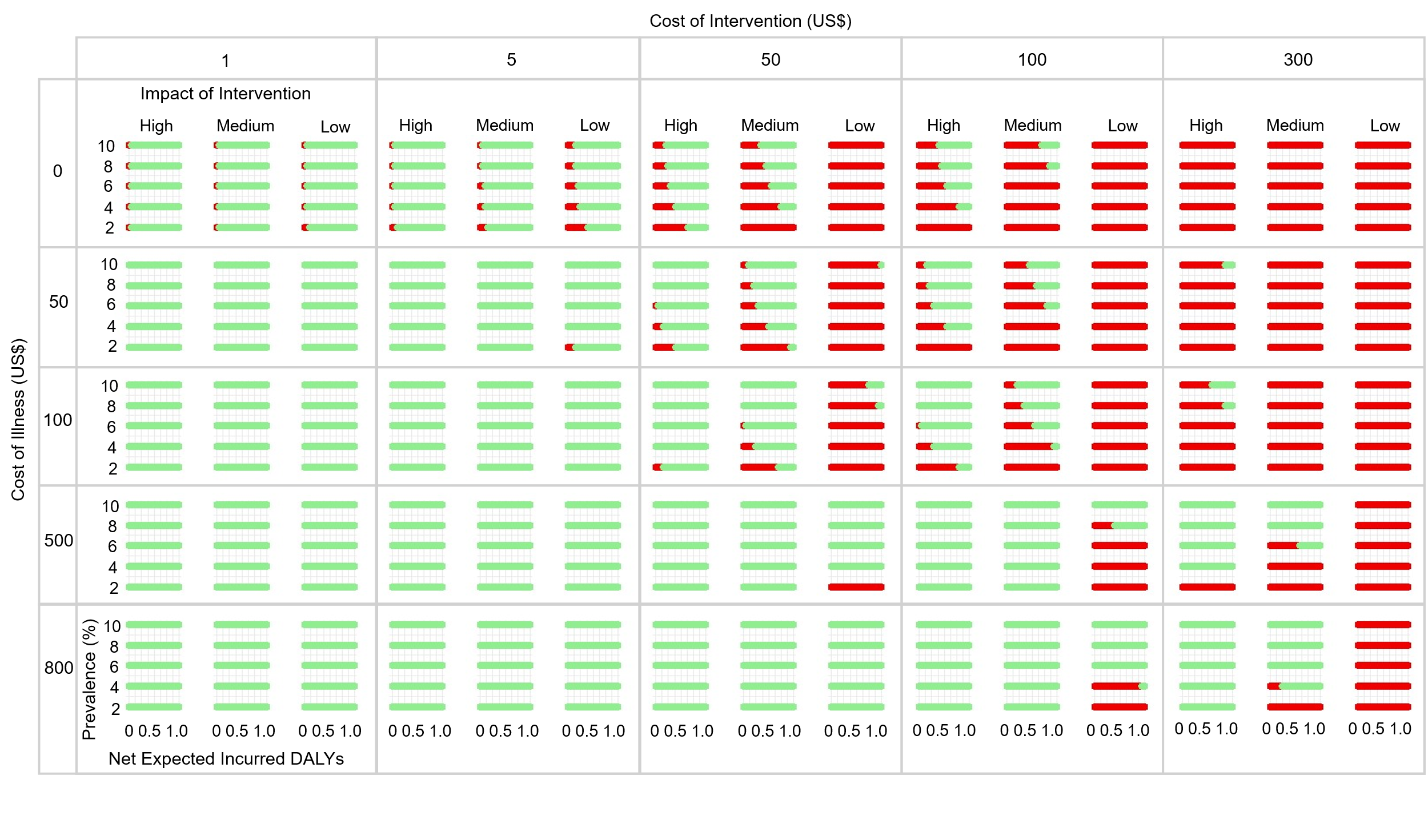
**Supporting Figure 1A: The 5D framework using a CET of US$ 200.**


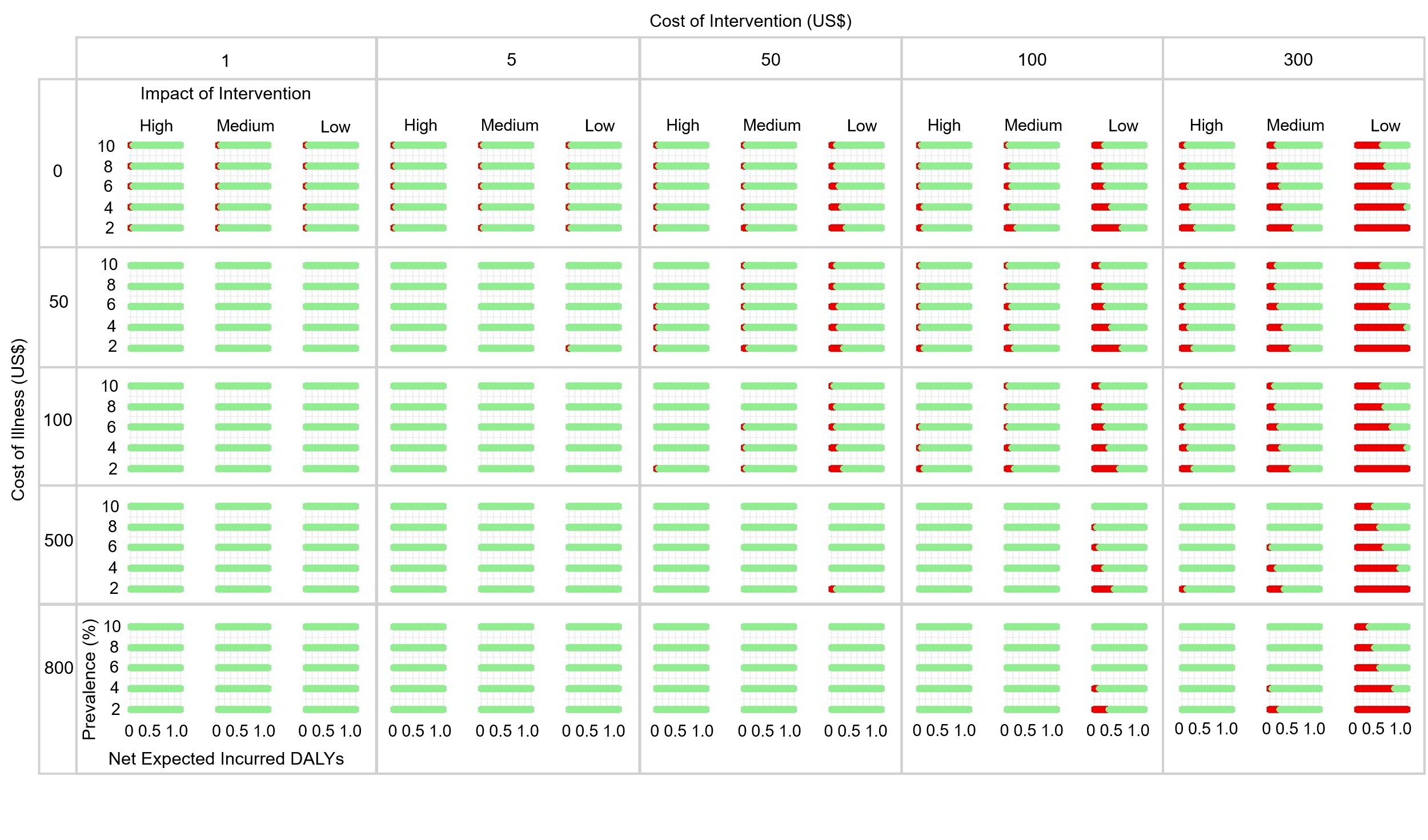
**Supporting Figure 1B: The 5D framework using a CET of US$ 3000.**


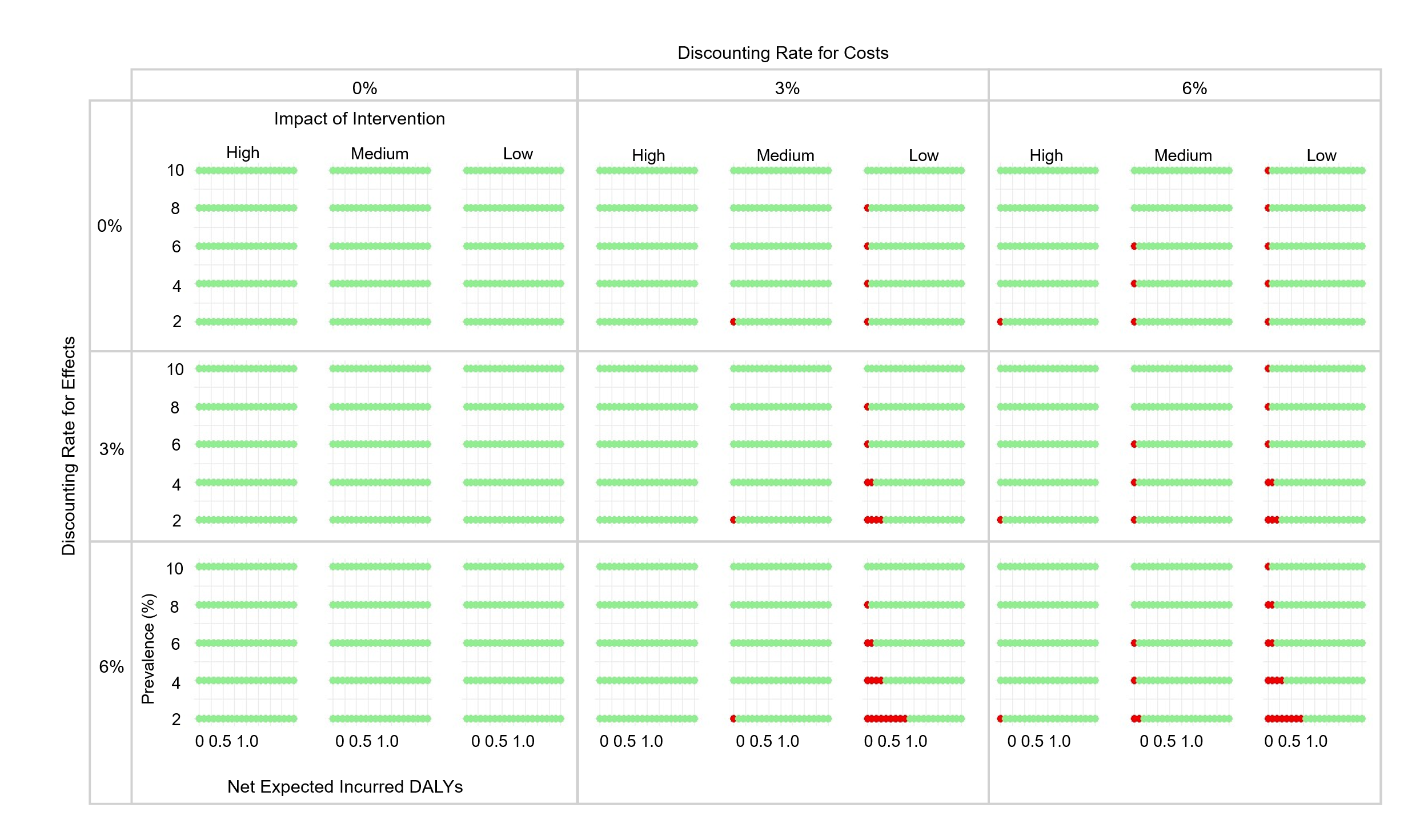


**Supporting Figure 2: Effect of varying discounting rates for costs and effects on the NHB values:** *based on a* *cost of illness of US$ 50 and a cost of intervention of US$ 5.*


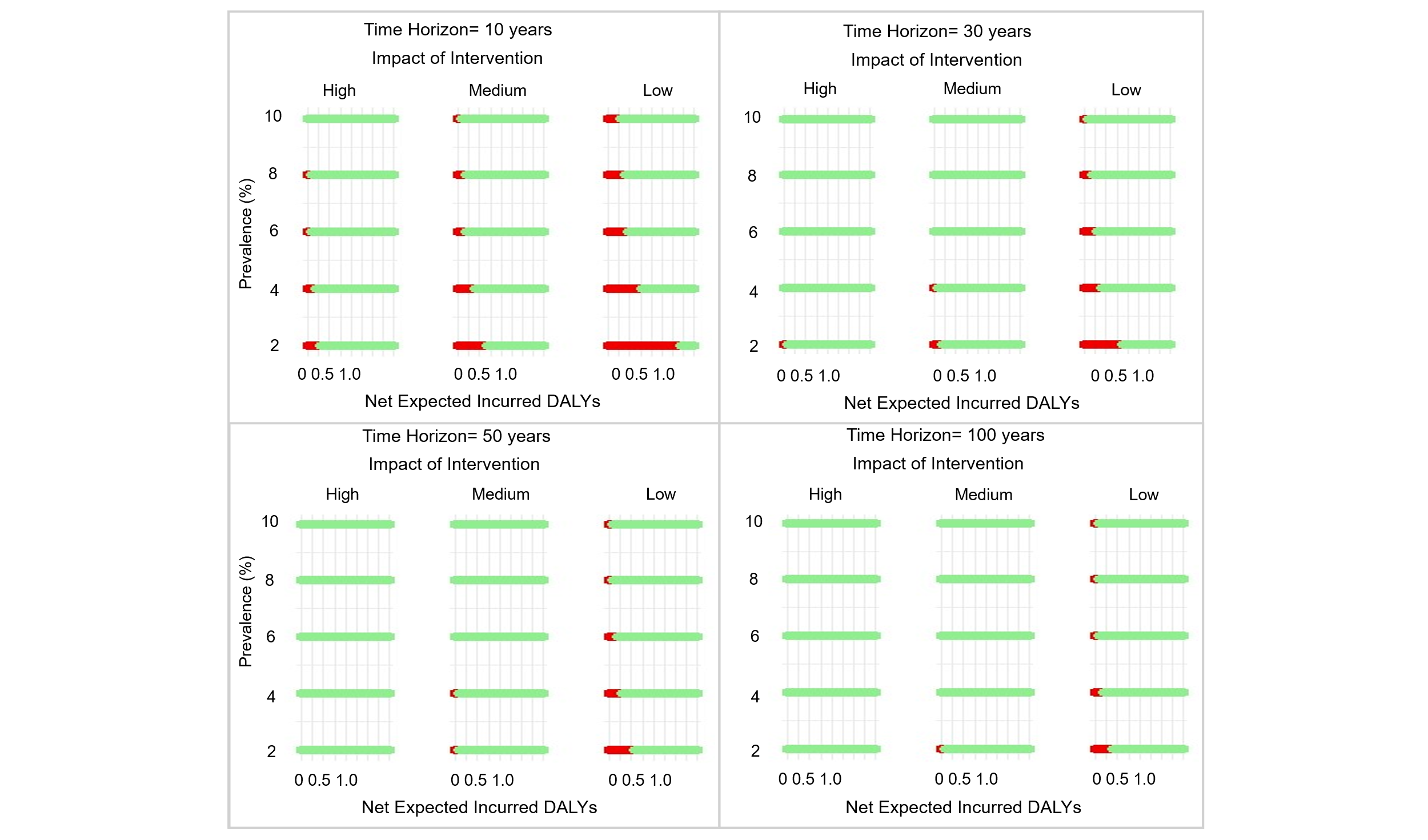


**Supporting Figure 3: The framework at different time horizons:** *based on a CET of US$ 800 and constant cost of illness of US$ 50 and cost of intervention of US$ 5, and discounting both costs and effects at 3%.*
